# Supplementary material for: A Genome-Wide Association Study of 2304 Extreme Longevity Cases Identifies Novel Longevity Variants
Source: Int J Mol Sci. 2022 Dec 21;24(1):116. doi: 10.3390/ijms24010116 (PMC9820206; doi:10.3390/ijms24010116)
Supplement: Supplementary file 1 [file ijms-24-00116-s001.zip › ijms-2073171-supplementary.pdf]

Supplementary Information

Additional Quality Control Filters

There were two batches (old and new) in the genotype data in the New England Centenarian Study (NECS) that used two different types of Illumina SNP arrays. In addition, to improve the statistical power of the current study and past studies, an additional set of approximately 3,500 controls from the Illumina control database genotyped using a variety of Illumina SNP arrays were added to the control sets of the NECS. These controls were of European ethnicity as validated by genome-wide principal component analysis and were used in prior analyses including references 7 and 8. To analyze a clean set of variants and remove errors due to different array technology, we defined the following three quantities:

1) Δ_1 = difference between the coded allele frequencies (CAF) in the NECS cases and controls
2) Δ_2 = difference between CAFs in the NECS cases in the old batch and NECS cases in the new batch
3) Δ_3 = difference between CAFs in the NECS controls and Illumina controls.

Based on these three quantities, we decided to eliminate SNPS if either

(1) $\Delta_2 > \Delta_1$ or
(2) $\Delta_3 > \Delta_1$.

Our rationale for this selection was that if a SNP satisfies (1) or (2), then the difference between CAF due to array technology can mask real effects and inflate the false positive rate.

Overview of the GWAS Pipeline

The current analysis used the GWAS pipeline developed by Song et al.[22], which integrates the steps of quality control, estimation of genome-wide principal components and full genetic relationship matrix, and association testing at the genome-wide level. The pipeline is implemented in Nextflow, a portable, scalable and parallelizable reactive workflow framework for data-intensive pipelines. The pipeline can be cloned from https://github.com/montilab/nf-gwas-pipeline, which also has step-by-step instructions at each process of the pipeline. We also provide vignettes to the two main R packages (SNPRelate[55] and GENESIS[56]) used in the pipeline:

SNPRelate:
http://bioconductor.riken.jp/packages/3.3/bioc/vignettes/GENESIS/inst/doc/pcair.html

GENESIS:
http://bioconductor.riken.jp/packages/3.3/bioc/vignettes/GENESIS/inst/doc/assoc_test.html

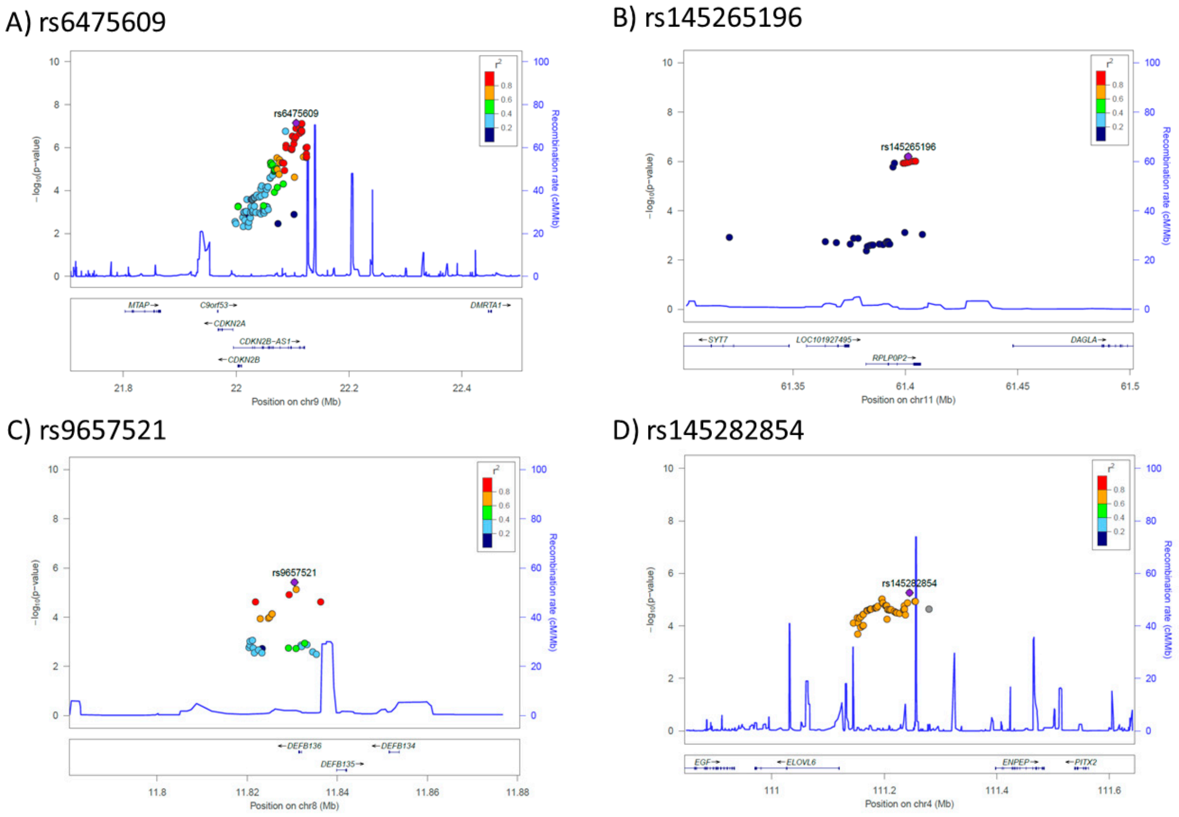

Supplementary Figure S1. Regional association plots for each of the 4 locus identified
